# Editorial: Acute heart failure: From bench to bedside

**DOI:** 10.3389/fphys.2023.1194213

**Published:** 2023-04-19

**Authors:** Alexander E. Berezin, Ioana Mozos, Michael Lichtenauer

**Affiliations:** ^1^ Internal Medicine Department, State Medical University, Zaporozhye, Ukraine; ^2^ Department of Internal Medicine II, Division of Cardiology, Paracelsus Medical University Salzburg, Salzburg, Austria; ^3^ Department of Functional Sciences–Pathophysiology, Center for Translational Research and Systems Medicine, “Victor Babes” University of Medicine and Pharmacy, Timisoara, Romania

**Keywords:** heart failure, biomarkers, stratification, prediction, risk scores

Acute heart failure (AHF) remains one of the leading cause of hospital admission among patients with known cardiovascular diseases (CVD) (Emmons-Bell et al., 2022). The levels of hospital mortality as well as 30-day-mortality and 1-year mortality after discharge in AHF patients with different congestion/perfusion status retain to be unacceptably high regardless of clear understanding of etiology and pathogenesis of the condition (Chioncel et al., 2019). Current guidelines, which are reported by American Heart Association/American College of Cardiology and European Cardiology Society, have been widely implemented in routine clinical praxis. However, there are several challenges regarding stratification of the CVD patients at higher risk of *de novo* AHF, prediction of both short-term and long-term prognosis and clinical trajectory of AHF, choice of optimal management at every stage of natural evolution of heart failure, role of circulating biomarkers in prognosis and management (Harjola et al., 2017; Al-Sadawi et al., 2022; McDonagh et al., 2022). Meanwhile, a role of cardiovascular and metabolic comorbidities, age and gender in *de novo* AHF and acute decompensated chronic heart failure (HF) retains to be not fully clear. Indeed, there is a large amount of clinical studies dedicated to a link between key pathogenetic mechanisms in any phenotypes of chronic HF, a risk of HF decompensation and cardiac/non-cardiac comorbidities (Harjola et al., 2017; Al-Sadawi et al., 2022).

In the four papers of this Research Topic various aspects of heart failure are discussed. In the first paper, Keir et al. focused on the prognostic value of an augmented peripheral chemo-reflex hypoxic ventilatory response as measured from brief hypoxic exposures in HF patients with preserved (HFpEF) and reduced (HFrEF) ejection fraction. They found that transient hypoxic ventilatory response tests exerted high capability of identifying differences in peripheral chemo-reflex sensitivity among HFpEF patients. The authors proposed that this methods may be addressed to further longitudinal studies to identify HFrEF patients with plausible benefit from carotid body intervention.

The results of the prospective observational study provided by Agra-Bermejo et al. yielded that the combination of adipocytokine omentin with anti-inflammatory properties and the acute-phase inflammatory protein orosomucoid known as α-1-acid–glycoprotein exhibited higher discriminative value than NT-proBNP, omentin and orosomucoid alone for re-admission and/or death in patients with *de novo* AHF. This study illustrates not only It suggests an independent predictive value for omentin and orosomucoid for AHF, and maybe but also plausible role of help in improving AHF outcomes during management with sodium-glucose co-transporter 2 inhibitors, which present anti-inflammatory properties. In the second study Li et al. investigated the association between acute kidney injury and adverse cardiac remodeling in a large patient population (*n* = 11573 patients with CVD, diabetes mellitus, chronic HF and chronic kidney disease, who were treated with undergone coronary angiography. The authors established that patients with acute kidney injury had a significantly higher risk of adverse cardiac remodeling when compared with those without acute kidney injury. Thus, the authors linked a the risk of HF due to left ventricular dysfunction with to kidney dysfunction after conventional coronary angiography. Taking into consideration a large amount of these procedures, a significance of the findings appears to be extremely important. In the third study, Budde et al. performed a narrative review which they thoroughly elucidated innate molecular mechanisms associated with systemic inflammation and oxidative stress regarding the nature of with a focus on the contribution of abundant cellular responses to HFpEF development. The authors discussed a role of age, gender, cardiac and non-cardiac comorbidities including diabetes mellitus, obesity along with adipose tissue dysfunction in the development of HFpEF. The authors emphasized that clustering of risk factors for HFpEF has hitherto not drawn much attention in clinical trials.
